# Bis(3-ammonio­methyl­pyridinium) cyclo­tetra­phosphate

**DOI:** 10.1107/S1600536810008056

**Published:** 2010-03-10

**Authors:** Hanène Hemissi, Mohammed Rzaigui, Salem S. Al-Deyab

**Affiliations:** a Laboratoire de Chimie des Matériaux, Faculté des Sciences de Bizerte, 7021 Zarzouna Bizerte, Tunisia; b Petrochemical Research Chair, College of Science, King Saud University, Riyadh, Saudi Arabia

## Abstract

In the title compound, 2C_6_H_10_N_2_^2+^·P_4_O_12_^4−^, which involves a doubly protonated 3-ammonio­methyl­pyridinium cation and a cyclo­tetra­phosphate anion, the cyclo­tetra­phospho­ric ring is arranged around an inversion center and the organic entity alternates with it, forming hybrid ribbons parallel to the *b* axis. The crystal structure is stabilized by a three-dimensional network of N—H⋯O and weaker C—H⋯O hydrogen bonds.

## Related literature

For properties of hybrid materials, see: Aakeröy *et al.*(1989 ▶); Sankar *et al.* (1993 ▶); Teraski *et al.* (1987 ▶); Vaughan (1993 ▶); Centi (1993 ▶); Ozin (1992 ▶). For related structures containing phospho­ric acid rings, see: Aloui *et al.* (2003 ▶); Hemissi *et al.* (2005 ▶); Averbuch-Pouchot & Durif (1991 ▶); Durif (1995 ▶). For bond lengths in pyridine, see: Bak *et al.* (1959 ▶). For hydrogen bonding, see: Blessing (1986 ▶); Brown (1976 ▶); Soumhi & Jouini (1996 ▶). Cyclo­tetra­phospho­ric acid was produced from Na_4_P_4_O_12_·4H_2_O, which was prepared according to the Ondik (1964 ▶) process.
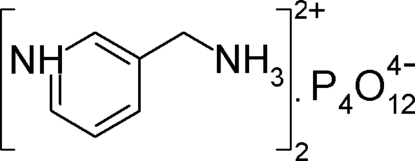


## Experimental

### 

#### Crystal data

2C_6_H_10_N_2_^2+^·P_4_O_12_^4−^
*M* *_r_* = 536.20
Triclinic, 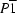

*a* = 7.849 (2) Å
*b* = 8.384 (2) Å
*c* = 9.448 (2) Å
α = 113.24 (2)°
β = 98.73 (3)°
γ = 108.76 (3)°
*V* = 512.4 (2) Å^3^
*Z* = 1
Ag *K*α radiation
λ = 0.56083 Å
μ = 0.23 mm^−1^
*T* = 293 K
0.35 × 0.3 × 0.15 mm

#### Data collection

Enraf–Nonius CAD-4 diffractometer
7923 measured reflections
5005 independent reflections
3963 reflections with *I* > 2σ(*I*)
*R*_int_ = 0.012
2 standard reflections every 120 min  intensity decay: 1%

#### Refinement

*R*[*F* ^2^ > 2σ(*F* ^2^)] = 0.034
*wR*(*F* ^2^) = 0.101
*S* = 1.08
5005 reflections
145 parameters
H-atom parameters constrained
Δρ_max_ = 0.49 e Å^−3^
Δρ_min_ = −0.47 e Å^−3^

### 

Data collection: *CAD-4 EXPRESS* (Enraf–Nonius, 1994 ▶); cell refinement: *CAD-4 EXPRESS*; data reduction: *XCAD4* (Harms & Wocadlo, 1996 ▶); program(s) used to solve structure: *SHELXS97* (Sheldrick, 2008 ▶); program(s) used to refine structure: *SHELXL97* (Sheldrick, 2008 ▶); molecular graphics: *ORTEP-3 for Windows* (Farrugia, 1997 ▶); software used to prepare material for publication: *WinGX* (Farrugia, 1999 ▶).

## Supplementary Material

Crystal structure: contains datablocks I, global. DOI: 10.1107/S1600536810008056/dn2541sup1.cif

Structure factors: contains datablocks I. DOI: 10.1107/S1600536810008056/dn2541Isup2.hkl

Additional supplementary materials:  crystallographic information; 3D view; checkCIF report

## Figures and Tables

**Table 1:** Hydrogen-bond geometry (Å, °)

*D*—H⋯*A*	*D*—H	H⋯*A*	*D*⋯*A*	*D*—H⋯*A*
N1—H1⋯O6	0.86	1.77	2.6294 (18)	175
N2—H2*A*⋯O5^i^	0.89	1.88	2.7079 (17)	154
N2—H2*B*⋯O3^ii^	0.89	2.02	2.7350 (17)	137
N2—H2*C*⋯O1^iii^	0.89	2.08	2.831 (2)	141
C1—H1*A*⋯O6^iv^	0.93	2.55	3.381 (2)	149
C4—H4⋯O5^v^	0.93	2.48	3.281 (2)	144
C5—H5⋯O4	0.93	2.60	3.256 (2)	128
C6—H6*B*⋯O1^i^	0.97	2.44	3.117 (2)	127

Symmetry codes: (i) 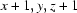; (ii) 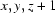; (iii) 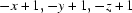; (iv) 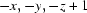; (v) 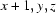.

**Fig. 1.**
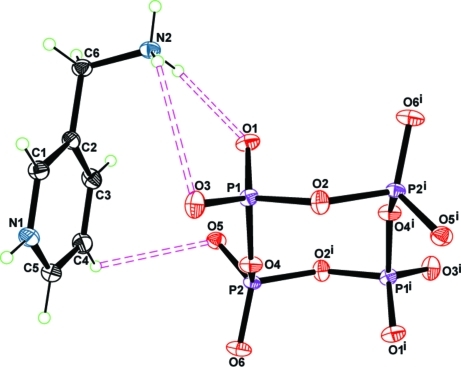
An ORTEP view of (I) with the atom-labelling scheme. Displacement ellipsoids are drawn at the 30% probability level. H atoms are represented as small spheres of arbitrary radii. [Symmetry codes: (i) 1 - x, 1 - y, 1 - z].

**Fig. 2.**
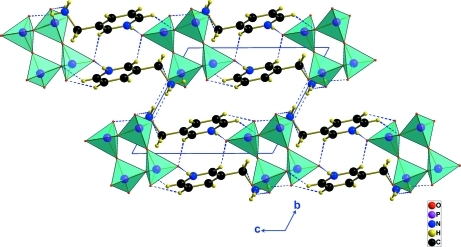
Projection of (I) along a axis.

## supplementary crystallographic information

### Comment

Hybrid materials with organic and inorganic components continue to be a focus area in solid state chemistry and material sciences due to their potential applications in various fields, such as nonlinear optics (Aakeröy *et al.*, 1989), heterogeneous catalysis (Centi, 1993), photochemical and photophysical process (Ozin, 1992), molecular sieves (Vaughan, 1993), ceramic precursors (Sankar *et al.*, 1993) and other areas that include electronic materials (Teraski *et al.*, 1987). In the present paper, the results of the x-ray structure analysis of a new organic cyclotetraphosphate, bis(3-ammoniomethylpyridinium) cyclotetraphosphate, are discussed with respect to the geometry and flexibility of the cyclotetraphosphate ring system and H-bonding interactions between the inorganic acceptor and the organic donor molecules.

The chemical composition of the title compound (I) includes two fundamental entities, the P_4_O_12_^4-^ ring and the organic cations (C_6_H_10_N_2_)^2+^. The geometrical configuration of these entities is depicted in Figure 1, while the complete atomic arrangement is shown in Figure 2. This latter shows that the crystal structure of (C_6_H_10_N_2_)_2_P_4_O_12_ can be described by hybrid ribbons where the organic and inorganic species are alternated. These ribbons, extended in the *b*-direction, are also connected between them in the two other directions via H-bonds to develop a three-dimensional network. The P_4_O_12_ rings are located around the inversion center (0, 0, 0) and are built up by only two independent PO_4_ tetrahedra. The P—P—P angles are 84.43 (1) and 95.57 (1)° and show that the tetramembered phosphoric rings are distorted in comparison with the ideal value (90°). It should be noted that such deviations are commonly observed in cyclotetraphosphoric ring anions with low internal symmetry as (I) (Aloui *et al.*, 2003; Hemissi *et al.*, 2005). Nevertheless, this distortion is comparatively less important than that observed in the hexamembered P_6_O_18_ rings (93.2 - 145.5°) (Averbuch-Pouchot & Durif, 1991). Consequently, P_4_O_12_ is less flexible than the P_6_O_18_ what could explain the pronounced distortion observed for the big rings compared with their smaller rings analogues. In spite of this distortion, the examination of the main geometrical feature of PO_4_ tetrahedra (P—O distances and P—O—P angles) shows that they are in accordance with values generally observed in condensed phosphates (Durif, 1995).

One crystallographically independent organic group exists in the asymmetric unit. Inside this organic molecule, both nitrogen atoms are protonated and so it is formulated (C_6_H_10_N_2_)^2+^. The examination of pyridinium ring shows that this unit is essentially planar with mean deviation of ±0.0036 Å from least-square plane defined by the six constituent atoms. The average C—N distances in pyridinium ring is 1.337 (2) Å and of the C—C bond lengths is 1.384 (2) Å. The latter value, being shorter than 1.39-1.41 Å, reported for non-substituent pyridine, may indicate some aromatic bond characters (Bak *et al.*, 1959). The pyridinium ring is non coplanar with its methylamine substituent (-CH_2_—NH_3_) which is evidenced by the torsion angle value of (C1—C2—C6—N2) equal to 96.29 (2)°. In addition to electrostatic and van der Waals interactions, the structure is further stabilized with a three-dimensional network of N—H···O and the weaker C—H···O hydrogen bonds (Table 1, Fig. 2)). In the hydrogen-bond scheme two main points should be noticed: (i) there is a bridging oxygen atom (O4) of the P_4_O_12_ ring involved in hydrogen bond and so that is rarely observed in organic condensed phosphates. Indeed, it was only observed in (C_6_H_10_N_2_)_2_P_4_O_12_.2H_2_O (Soumhi *et al.*, 1996). (ii) Inside the structure, there are two strong hydrogen bonds with N···O distances equal to 2.629 (2) and 2.708 (2) Å. The others are weaker within N(C)···O distances falling from 2.735 (2) to 3.381 (2) Å (Brown, 1976; Blessing, 1986).

### Experimental

Crystals of the title compound were prepared by adding ethanolic solution (5 ml) of 3-aminopicolamine (11.04 mmol) dropwise to an aqueous solution of cyclotetraphosphoric acid (5.52 mmol, 20 ml). Good quality of colourless prisms were obtained after a slow evaporation during few days at ambient temperature. The cyclotetraphosphoric acid H_4_P_4_O_12_, was produced from Na_4_P_4_O_12_.4H_2_O, prepared according to the Ondik process (Ondik, 1964), through an ion-exchange resin in H-state (Amberlite IR 120).

### Refinement

All H atoms were positioned geometrically and treated as riding on their parent atoms, [N–H = 0.89, C–H =0.96 Å (CH_3 _) with with *U*_iso_(H) = 1.5Ueq and C–H =0.96 Å (Ar–H), with *U*_iso_(H) = 1.5Ueq]

### Crystal data

**Table:**

2C_6_H_10_N_2_^2+^·P_4_O_12_^4^^−^	*Z* = 1
*M**_r_* = 536.20	*F*(000) = 276
Triclinic, *P*1	*D*_x_ = 1.738 Mg m^−^^3^
Hall symbol: -P 1	Ag *K*α radiation, λ = 0.56083 Å
*a* = 7.849 (2) Å	Cell parameters from 25 reflections
*b* = 8.384 (2) Å	θ = 9.0–10.7°
*c* = 9.448 (2) Å	µ = 0.23 mm^−^^1^
α = 113.24 (2)°	*T* = 293 K
β = 98.73 (3)°	Prism, colourless
γ = 108.76 (3)°	0.35 × 0.3 × 0.15 mm
*V* = 512.4 (2) Å^3^	

### Data collection

**Table:**

Enraf–Nonius CAD-4 diffractometer	*R*_int_ = 0.012
Radiation source: Enraf–Nonius FR590	θ_max_ = 28.0°, θ_min_ = 2.2°
graphite	*h* = −13→13
non–profiled ω scans	*k* = −14→14
7923 measured reflections	*l* = −6→15
5005 independent reflections	2 standard reflections every 120 min
3963 reflections with *I* > 2σ(*I*)	intensity decay: 1%

### Refinement

**Table:**

Refinement on *F*^2^	Primary atom site location: structure-invariant direct methods
Least-squares matrix: full	Secondary atom site location: difference Fourier map
*R*[*F*^2^ > 2σ(*F*^2^)] = 0.034	Hydrogen site location: inferred from neighbouring sites
*wR*(*F*^2^) = 0.101	H-atom parameters constrained
*S* = 1.08	*w* = 1/[σ^2^(*F*_o_^2^) + (0.049*P*)^2^ + 0.1514*P*] where *P* = (*F*_o_^2^ + 2*F*_c_^2^)/3
5005 reflections	(Δ/σ)_max_ = 0.001
145 parameters	Δρ_max_ = 0.49 e Å^−^^3^
0 restraints	Δρ_min_ = −0.47 e Å^−^^3^

### Special details

**Table:**

Geometry. All esds (except the esd in the dihedral angle between two l.s. planes) are estimated using the full covariance matrix. The cell esds are taken into account individually in the estimation of esds in distances, angles and torsion angles; correlations between esds in cell parameters are only used when they are defined by crystal symmetry. An approximate (isotropic) treatment of cell esds is used for estimating esds involving l.s. planes.
Refinement. Refinement of *F*^2^ against ALL reflections. The weighted *R*-factor wR and goodness of fit *S* are based on *F*^2^, conventional *R*-factors *R* are based on *F*, with *F* set to zero for negative *F*^2^. The threshold expression of *F*^2^ > σ(*F*^2^) is used only for calculating *R*-factors(gt) etc. and is not relevant to the choice of reflections for refinement. *R*-factors based on *F*^2^ are statistically about twice as large as those based on *F*, and *R*- factors based on ALL data will be even larger.

### Fractional atomic coordinates and isotropic or equivalent isotropic displacement parameters (Å^2^)

**Table:**

	*x*	*y*	*z*	*U*_iso_*/*U*_eq_	
O4	0.14028 (12)	0.17020 (14)	0.16858 (12)	0.02591 (17)	
O2	0.20484 (12)	0.04327 (13)	−0.08942 (12)	0.02816 (18)	
O1	0.07961 (14)	0.30061 (15)	−0.02599 (13)	0.03096 (19)	
O3	0.41615 (12)	0.37060 (14)	0.12629 (14)	0.0328 (2)	
O5	−0.09804 (14)	0.30451 (14)	0.25390 (12)	0.02842 (18)	
O6	0.01424 (14)	0.12073 (17)	0.37735 (13)	0.0330 (2)	
P1	0.21242 (4)	0.23999 (4)	0.04410 (4)	0.02125 (7)	
P2	−0.03708 (4)	0.15037 (4)	0.23497 (4)	0.02013 (7)	
N2	0.71399 (14)	0.34670 (15)	1.01546 (13)	0.02438 (18)	
H2A	0.7405	0.3187	1.0954	0.037*	
H2B	0.6259	0.3920	1.0259	0.037*	
H2C	0.8188	0.4345	1.0204	0.037*	
N1	0.37134 (15)	0.21366 (16)	0.52122 (14)	0.0284 (2)	
H1	0.2559	0.1902	0.4771	0.034*	
C1	0.41069 (16)	0.17716 (18)	0.64460 (16)	0.0259 (2)	
H1A	0.3140	0.1291	0.6824	0.031*	
C4	0.6907 (2)	0.3225 (2)	0.53050 (17)	0.0320 (3)	
H4	0.7847	0.3727	0.4913	0.038*	
C6	0.64164 (17)	0.17070 (18)	0.85588 (16)	0.0264 (2)	
H6A	0.5287	0.0744	0.8515	0.032*	
H6B	0.7369	0.1203	0.8456	0.032*	
C2	0.59489 (16)	0.21046 (16)	0.71693 (14)	0.02293 (19)	
C5	0.5057 (2)	0.28559 (19)	0.46376 (16)	0.0309 (2)	
H5	0.4735	0.3106	0.3784	0.037*	
C3	0.73527 (17)	0.28345 (19)	0.65771 (16)	0.0288 (2)	
H3	0.8598	0.3064	0.7034	0.035*	

### Atomic displacement parameters (Å^2^)

**Table:**

	*U*^11^	*U*^22^	*U*^33^	*U*^12^	*U*^13^	*U*^23^
O4	0.0190 (3)	0.0374 (5)	0.0315 (4)	0.0132 (3)	0.0114 (3)	0.0233 (4)
O2	0.0190 (3)	0.0275 (4)	0.0306 (4)	0.0079 (3)	0.0068 (3)	0.0089 (3)
O1	0.0281 (4)	0.0376 (5)	0.0413 (5)	0.0170 (4)	0.0131 (4)	0.0284 (4)
O3	0.0174 (3)	0.0290 (4)	0.0409 (5)	0.0034 (3)	0.0105 (3)	0.0109 (4)
O5	0.0331 (4)	0.0325 (4)	0.0273 (4)	0.0199 (4)	0.0114 (4)	0.0157 (4)
O6	0.0267 (4)	0.0532 (6)	0.0328 (5)	0.0175 (4)	0.0104 (4)	0.0320 (5)
P1	0.01556 (11)	0.02324 (13)	0.02726 (14)	0.00693 (9)	0.00856 (10)	0.01444 (11)
P2	0.01707 (11)	0.02625 (13)	0.02125 (13)	0.00966 (10)	0.00632 (9)	0.01460 (11)
N2	0.0213 (4)	0.0309 (5)	0.0267 (4)	0.0122 (3)	0.0078 (3)	0.0180 (4)
N1	0.0235 (4)	0.0314 (5)	0.0285 (5)	0.0109 (4)	0.0019 (4)	0.0152 (4)
C1	0.0198 (4)	0.0305 (5)	0.0293 (5)	0.0101 (4)	0.0065 (4)	0.0167 (5)
C4	0.0303 (6)	0.0337 (6)	0.0281 (6)	0.0080 (5)	0.0117 (5)	0.0146 (5)
C6	0.0250 (5)	0.0289 (5)	0.0287 (5)	0.0123 (4)	0.0054 (4)	0.0172 (4)
C2	0.0197 (4)	0.0245 (5)	0.0235 (5)	0.0091 (4)	0.0047 (4)	0.0114 (4)
C5	0.0360 (6)	0.0298 (6)	0.0248 (5)	0.0116 (5)	0.0055 (5)	0.0143 (5)
C3	0.0201 (4)	0.0345 (6)	0.0284 (6)	0.0092 (4)	0.0073 (4)	0.0137 (5)

### Geometric parameters (Å, °)

**Table:**

O4—P2	1.5992 (10)	N1—C5	1.3393 (19)
O4—P1	1.6057 (10)	N1—H1	0.8600
O2—P2^i^	1.6044 (14)	C1—C2	1.3875 (17)
O2—P1	1.6085 (11)	C1—H1A	0.9300
O1—P1	1.4766 (10)	C4—C5	1.372 (2)
O3—P1	1.4781 (12)	C4—C3	1.389 (2)
O5—P2	1.4739 (10)	C4—H4	0.9300
O6—P2	1.4824 (10)	C6—C2	1.4990 (17)
P2—O2^i^	1.6044 (14)	C6—H6A	0.9700
N2—C6	1.4894 (18)	C6—H6B	0.9700
N2—H2A	0.8900	C2—C3	1.3875 (18)
N2—H2B	0.8900	C5—H5	0.9300
N2—H2C	0.8900	C3—H3	0.9300
N1—C1	1.3345 (17)		
			
P2—O4—P1	136.26 (6)	C5—N1—H1	118.9
P2^i^—O2—P1	134.03 (6)	N1—C1—C2	120.46 (12)
O1—P1—O3	120.41 (7)	N1—C1—H1A	119.8
O1—P1—O4	110.99 (6)	C2—C1—H1A	119.8
O3—P1—O4	106.65 (6)	C5—C4—C3	118.82 (13)
O1—P1—O2	111.42 (7)	C5—C4—H4	120.6
O3—P1—O2	105.82 (7)	C3—C4—H4	120.6
O4—P1—O2	99.36 (6)	N2—C6—C2	111.53 (10)
O5—P2—O6	119.04 (7)	N2—C6—H6A	109.3
O5—P2—O4	112.23 (6)	C2—C6—H6A	109.3
O6—P2—O4	105.42 (6)	N2—C6—H6B	109.3
O5—P2—O2^i^	106.33 (6)	C2—C6—H6B	109.3
O6—P2—O2^i^	109.45 (7)	H6A—C6—H6B	108.0
O4—P2—O2^i^	103.28 (6)	C3—C2—C1	117.98 (11)
C6—N2—H2A	109.5	C3—C2—C6	120.87 (11)
C6—N2—H2B	109.5	C1—C2—C6	121.15 (11)
H2A—N2—H2B	109.5	N1—C5—C4	120.20 (12)
C6—N2—H2C	109.5	N1—C5—H5	119.9
H2A—N2—H2C	109.5	C4—C5—H5	119.9
H2B—N2—H2C	109.5	C2—C3—C4	120.36 (12)
C1—N1—C5	122.18 (11)	C2—C3—H3	119.8
C1—N1—H1	118.9	C4—C3—H3	119.8

Symmetry codes: (i) −*x*, −*y*, −*z*.

### Hydrogen-bond geometry (Å, °)

**Table:**

*D*—H···*A*	*D*—H	H···*A*	*D*···*A*	*D*—H···*A*
N1—H1···O6	0.86	1.77	2.6294 (18)	175
N2—H2A···O5^ii^	0.89	1.88	2.7079 (17)	154
N2—H2B···O3^iii^	0.89	2.02	2.7350 (17)	137
N2—H2C···O1^iv^	0.89	2.08	2.831 (2)	141
C1—H1A···O6^v^	0.93	2.55	3.381 (2)	149
C4—H4···O5^vi^	0.93	2.48	3.281 (2)	144
C5—H5···O4	0.93	2.60	3.256 (2)	128
C6—H6B···O1^ii^	0.97	2.44	3.117 (2)	127

Symmetry codes: (ii) *x*+1, *y*, *z*+1; (iii) *x*, *y*, *z*+1; (iv) −*x*+1, −*y*+1, −*z*+1; (v) −*x*, −*y*, −*z*+1; (vi) *x*+1, *y*, *z*.
